# Knowing better, doing better: Taking stock of the UN Agenda 2030, its means, its progress, and its shortfalls

**DOI:** 10.1016/j.isci.2026.116925

**Published:** 2026-08-03

**Authors:** Rega Sota, Daniel M. Kammen, Magdalena Klemun, Sandra Venghaus

**Affiliations:** 1 Economics of Sustainability, School of Business and Economics, RWTH Aachen University, Aachen, Germany; 2 Department of Civil and Systems Engineering and School of Advanced International Studies, Johns Hopkins University, Baltimore, MD, USA; 3 Department of Civil and Systems Engineering, Johns Hopkins University, Baltimore, MD, USA; 4 Economics of Sustainability, School of Business and Economics, RWTH Aachen University and Jülich Systems Analysis, Institute of Climate and Energy Systems, Forschungszentrum Jülich, Jülich, Germany

**Keywords:** Sustainable Development Goals, UN 2030 Agenda, SDG monitoring, sustainability policy

## Abstract

With 4 years left until the deadline of the UN Agenda 2030, our article ascertains the urgency of appraising the role of the Sustainable Development Goals (SDGs). We consider their main shortcomings and suggest interventions to enhance the global impact of the SDGs and their succeeding framework. We highlight the limitations of a static document designed for the following 15 years amid a rapidly changing global landscape that consistently poses new challenges and demands the development of better responses. Our perspective calls for the need to embed periodic reviews in the Agenda, a decentralized approach to applying the SDGs and the introduction of enforceable commitments among signatory parties. An effective set of sustainability pledges would recognize the risk that the fragile transition away from fossil fuels poses to communities worldwide and support a mission-driven approach to financing solutions. In addition, the Agenda requires rigorous methods that quantify the impact of sustainability interventions.

## Graphical abstract

**Figure:**
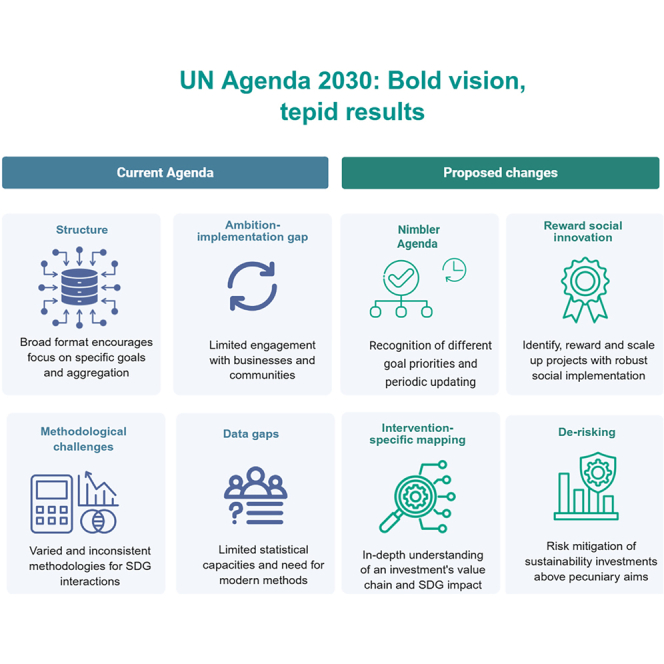

## Teaser

Global change; Environmental policy; Energy sustainability; Social sciences

## Introduction: Bold vision, tepid results

Each year, thousands of diplomats, business executives, scientists, and policy experts gather in attendance of the Conference of the Parties (COP) of the United Nations Framework Convention on Climate Change (UNFCC), marking a closely followed meeting by many others worldwide.^1^ Since 2015, COPs have focused regularly on the commitment to achieve net-zero energy systems by 2050 enshrined in the Paris Agreement. The 17 Sustainable Development Goals (SDGs) of UN Agenda 2030 have been around for just as long as the consensus to limit warming to 1.5°C above pre-industrial levels by the end of the century, yet have been the recipient of scarcer attention. Since the COPs represent a yearly occurrence when policy’s attention turns to sustainability challenges, the upcoming conference is a good opportunity to appraise the UN Agenda 2030. Given that the SDGs marked one decade since their launch, have 4 years left until their deadline, and are already the subject of discussions about the format of the UN goals to follow,^2^ we assert that a reflection on the SDGs needs to constitute a major topic of discussion at COP 2026 and, ideally, beyond in genuine efforts to turn words into action.

The UN Agenda 2030, more expansive than the Millennium Development Goals (MDGs), broadened the pursuit of sustainability to encompass both stewardship of natural resources, including water (SDG 6) and air quality (SDG 11), and enduring social challenges, including poverty eradication (SDG 1), food security (SDG 2), and gender equality (SDG 5). Crucially, it recognizes the interdependence between goals.^3^ Yet, operationalization of the SDGs has lagged behind their innovative vision of sustainability governance by goal-setting.^4^ We argue in this perspective that the underwhelming results of the SDGs stem from a fundamental mismatch between their rigid, broad, and checklist-based structure and the decentralized approach that is urgently needed, and in some instances already taking place, to ensure their progress. Between wanting to preserve the practical and compact format of the eight MDGs and an expansive vision of sustainability that extends far beyond them, a tenuous relationship has developed. By expanding their scope in both aims and geography, the 17 SDGs, unlike the preceding health-focused MDGs, have delved into areas where “best practices” are not yet established, uncertainty is significant, and knowledge about complementarities between goals remains incomplete. Yet, the ability of the Agenda to prescribe courses of action in response to mounting and rapidly shifting challenges remains feeble.

We develop this argument based on the literature and policy debates on key shortcomings of the Agenda over the past decade and outline the role played by the structure and content of the Agenda in limiting the progress of the SDGs. Our analysis emphasizes four areas for development: (1) a more adaptive and prioritized Agenda that can respond to changing sustainability challenges and varying local contexts; (2) the incorporation of rigorous, intervention-focused quantitative frameworks that allow policymakers to assess the impacts and trade-offs of concrete sustainability choices; (3) a mission-oriented approach to de-risking sustainability investments during periods of socio-technical transition, when market incentives alone may be insufficient; and (4) stronger recognition and support of decentralized, community-driven solutions and social innovations that can complement top-down governance approaches. Together, these recommendations span both the redesign of the SDG framework itself and the practical mechanisms required for its implementation, integrating rigorous evidence-based evaluation with technological, financial, and community-centered pathways for sustainable transformation.

We focus on policymakers, whether at the local, national, or international levels, as the target audience for our piece. While our perspective advocates for changes that would move the Agenda away from the exclusive domain of policymakers, we recognize that the framework has been influenced, since its conceptualization, by policy frames and decisions. We call on policy to institute the changes necessary for shifting from a discursive realm to an actionable one.

## SDG shortcomings

While criticism of the SDGs can take many forms, we have focused on the necessity to reframe the Agenda’s structure and enhance the rigorous operationalization and implementation of the goals.

1. Rethinking the Agenda’s format and size

The UN’s conceptualization of Agenda 2030 as a list of 17 goals and its implicit recognition of equal weight among the “indivisible and interconnected” SDGs has met with detractors.^5^^,^^6^ In response, scholars have sought to identify priority targets in national or regional contexts (see Weitz et al., Lyytimäki et al., and Bie et al.^7^^,^^8^^,^^9^). Some have called for the identification of “essential variables” for sustainable development,^10^ with a particular emphasis on poverty and inequality, and have criticized the UN framework for containing incompatibilities between indicators and targets^11^ or between targets and goals.^12^ Aggregate metrics (e.g., the SDG Index) have been devised to capture the status of all the SDGs, while the Agenda’s sprawling hierarchy has fueled demand by policymakers for analysis tools that reduce its complexity to “directly actionable” results, often with limited emphasis on methodological details.^13^ Despite receiving initial praise for its expanse, this feature of the Agenda is not preserved in the numerous quantitative methodologies proposed to analyze it and instead the complexity tends to be collapsed into single numerical results.^14^ These instances are a recognition that the Agenda is too broad for effective practical application and for enacting change.

Increasing the quantity and extent of aims called for in the name of sustainability might be beneficial in the short run but eventually leads to low marginal gains from integration and could even trigger the development of what Bolognesi and Nahrath^15^ call “transversal transaction costs” arising, for instance, from double penalties or contradictory clauses. This contrast prompts a reconsideration of the Agenda’s framing to meet standards of rigor while being less burdensome, particularly for subnational actors, to adopt.

2. Limited uptake of SDGs in business and policy communities

Bridging the gap between knowledge on the SDGs and the action evoked by their targets remains a challenge and has contributed to the Agenda’s limited global reach, with many SDGs stagnating or showing signs of regression by their mid-point assessment in 2023.^16^ Although some scholars maintain that the SDGs should only be regarded as rough guidelines for policymaking, this view invites ambiguity and weakens the UN Agenda’s ambition.^17^ The argument that the goals should remain broad signposts was called into question when the SDGs were complemented with quantitative indicators that were quickly embraced by the research community as soon as they were finalized in 2017.^18^ With the 2017 changes came a newfound belief that if the interactions between SDGs could be accurately quantified, it would be easier to trace paths toward achieving the sustainability vision that did not involve trade-offs between goals.

However, the discrepancies between aims and reality in the bold promises of the UN Agenda 2030 did not stop with the release of quantitative indicators. The aspiration-implementation gap is symptomatic of setbacks in the science-to-policy pipeline and indicative of an aversion to decentralization among institutions tasked with designing and implementing the SDGs. Compartmentalized and siloed expertise is still the norm across institutions. The different actors responsible for fulfilling particular SDGs rarely operate under the same jurisdiction, complicating the translation of the Agenda across national, regional, or urban scales.^19^

The gap between SDG aspirations and their implementation persists among companies as well. The overall engagement of the business sector with the SDGs remains largely symbolic, stemming from the Agenda’s non-binding nature, little involvement of businesses in crafting the SDGs, and a selective approach by companies in reporting goals.^20^ Despite ample scholarly research attesting to the benefits of firms’ sustainability and long-term visions for their valuation,^21^ the SDGs have not provided clear incentives to counter a prevalent myopic orientation among businesses.

Part of the responsibility for the limited uptake of the goals by businesses and local communities rests with the Agenda for invoking state governments as the main actors responsible for achieving the goals. Although having governments bear primary responsibility for global aims is common practice and often desirable by decision-makers,^22^ the Agenda has devoted less attention to mechanisms that connect these high-level commitments with the diverse sustainability initiatives already emerging from businesses, communities, and local institutions. The challenge is therefore not to replace top-down governance with decentralized implementation but to better connect these scales of action through a responsive governance model in which broad sustainability goals are coupled with locally adapted implementation, iterative learning, and the diffusion and adaptation of successful practices across contexts. Such a model would help avoid the coordination failures and unintended consequences that can arise when broad sustainability goals are translated into local action without sufficient feedback mechanisms.^23^

3. Lack of quantitative, intervention-focused studies on SDG linkages

The scientific community has explored SDG interactions through varied terminology and methods for a decade. The results and recommendations derived by the reported methods remain inconclusive and at times contradictory.^14^ The field is marred by a lack of consistency in the interpretation of interactions and a limited focus on the causal mechanisms at work between different SDGs. The preference of policymakers for simple terminology and easy-to-interpret results^13^ has compounded the interest in the early rudimentary methodologies for studying interlinkages. In contrast, promising quantitative frameworks for studying SDG interactions remain unexplored by decision-makers. While some large policy bodies have made spurious reference to the SDGs in decision-making, their use in policy, business, and community-level decisions trails behind.^24^ The absence of a unified and consistent framework for studying SDG interlinkages is a significant hurdle to their adoption.

Having no clear quantitative definitions to employ for SDG interactions and their spillovers hinders collaboration among different entities facing shared types of problems. It also prevents the application of penalties toward members making no effort at progress or actively undermining it. Thus, the cost of non-compliance is low and the goals are rendered moot. There is a need for what Sabel and Victor^22^ identify as “a stronger system of pledging.” This would imply devising a quantitatively robust framework to study interactions between goals and employing it to boost complementarities and minimize inter-goal conflicts. It would also imply the introduction of costs for not making progress toward the pledged goals.

Some argue that imposing penalties for the SDGs would hinder their adoption and thus slow progress overall. However, recent history has shown that if countries see concrete benefits in adoption and believable threats in the form of penalties, there is openness to negotiation. Conversely, relying on largely nominal promises that no one upholds has similarly been shown to devolve rapidly into tailor-made reciprocal agreements, which *de facto* invalidate international frameworks^25^^,^^26^ and undermine any global progress toward the goals. The time is ripe for a UN Agenda that comes with some degree of “skin in the game.”

4. Persistent data gaps

Measuring SDG progress and implementing effective feedback from SDG interventions to improve policies rely on data availability. Improving the quality and availability of SDG indicator data has been a recurring theme in discussions surrounding the implementation of the SDGs.^27^ The Inter-Agency Expert Group on SDGs has made improvements in the methodologies and data availability for SDGs; however, the financial and institutional capabilities to build and maintain fine-grained datasets remain unsatisfactory, particularly among the statistical bodies of developing nations.^28^ In 2025, UN language still defines “good data coverage” for SDG indicators as data being available in at least 50% of countries—an implicit recognition by the UN custodian agencies of significant gaps in coverage.^29^

Large-scale data mining and analytics, combined with non-traditional data sources such as mobile phones for socioeconomic information,^30^ offer a potential pathway toward improved real-time monitoring capabilities and SDG intervention analyses, especially in regions with limited statistical capabilities. However, this approach also poses challenges related to security, privacy, and capacity building, which are necessary to analyze large volumes of new, unstructured data. The non-traditional solutions available along with the distinct challenges they pose are another reason to embed a process of regular review within the UN Agenda such that SDG data gaps do not linger indefinitely and the dilemmas that come with applying innovative solutions are thoroughly examined.

## Proposed solutions

Our earlier analysis identified structural weaknesses in the format of the Agenda as well as problems that hinder the implementation of the SDGs. Motivated by this categorization, we propose solutions by following a similar structure.

1. A nimbler Agenda

Not all SDGs carry the same weight across different contexts. Differences are driven by both geography and context (not all communities have marine ecosystems to address under SDG 14) as well as by budgetary constraints that demand prioritization. Recognizing this, the UN Agenda must acknowledge the primacy of efforts to combat poverty and inequality^6^ given that limited progress on these goals hinders progress on other SDGs. As an illustrative case, several industrializing countries have made significant advancements in adopting green technologies, whether in the mobility sector or electricity generation. Yet, unregulated adoption by high- and middle-income groups in these countries has disproportionately harmed those least able to afford these technologies, as with the mass exodus from the national electricity grid by middle- and high-income farmers who could afford to install photovoltaics in Pakistan.^31^ By explicitly prioritizing the poverty and inequality SDGs on a global scale, the UN would incentivize countries to come up with well-crafted policies to prevent problematic rollouts of climate-beneficial technologies. As illustrated earlier, the fragility of the transition away from fossil-based systems poses new challenges,^32^ even as it lays out solutions, and would thus benefit from a compact agenda focused on a few absolute priorities.

Consistent with a nimbler style and wider embrace of bottom-up initiatives, a responsive Agenda would include clauses that call for periodic reviews of its structure and that trigger reconsideration of its content under changing circumstances.^2^^,^^33^ Much can change in a 15-year span and examples for the UN Agenda 2030 already include the COVID-19 pandemic and the booming AI and data center industries. More recently, the rise of AI and the disruptions with which affected communities are wrestling regarding the electricity grid, water supply, as well as the accompanying shifts in the labor market were not envisaged in 2015. As a result, the UN Agenda 2030, stuck in the realities of a decade ago, is weaker for it.

2. Intervention-centric, evidence-based methods for studying SDG interlinkages

**Figure 1:**
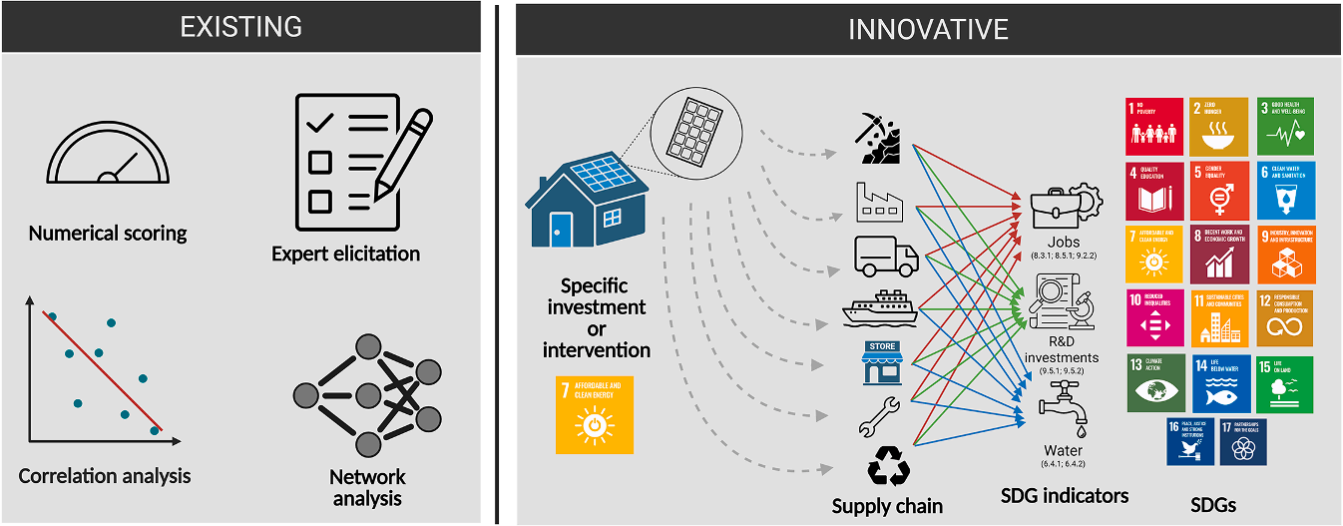
The contrast between current dominant interlinkage methods and an intervention-specific mapping focused on the whole product value chain by Klemun et al. (2023)

Earlier work on SDG interlinkages abstracts away from the quantifiable interventions that may strengthen, weaken, or create new linkages in practice (e.g., technology scaling, retrofitting, and decommissioning). Recent research has proposed a reexamination of SDG interlinkages by grounding SDG impact evaluations in specific interventions, including technology investment. For example, the mechanistic approach introduced by Klemun et al.^34^ begins by selecting an investment that directly supports a primary SDG (e.g., solar photovoltaic systems (PVs) for SDG 7) and then maps the industries involved in producing, deploying, operating, and decommissioning that technology. Each industry is, in turn, linked to various SDGs through its socio-economic or environmental impacts on specific SDG indicators. The two-step process, mediated by a specific technology, permits deducing potential linkages between the primary SDG and the remaining goals of the Agenda (Figure 1).

The mechanistic mapping (Figure 1) is promising for studying and quantifying SDG interactions for several reasons. It grounds the study of interlinkages in specific innovations and initiatives, as well as particular geographic locations. The most widespread interlinkage methodologies, especially those involving correlation,^35^^,^^36^ have largely avoided such specificity but simultaneously limited the use of SDGs in decision-making.^37^ The novel procedure described earlier requires an understanding of the technology’s supply chain rather than relying solely on the product’s end function. Moreover, while existing methods like life cycle assessment (LCA) produce results only mapped to some SDGs through their impact categories, mechanistic mapping tackles the entirety of the goals.^34^ If adopted by businesses, this method and similar methods could prompt greater scrutiny of value chains.^38^ In establishing links between relevant industries and the SDGs through step 2 in our procedure (Figure 1), robust quantitative methods extracted from the literature can be employed, thus accommodating existing frameworks that might have been deemed too complex for use in policy.^13^ Employing intervention-focused, data-driven methods at scale can enable a comparison of alternative technologies, allowing communities to select those most beneficial to them.

Methodologies that allow for mapping investments to the SDGs, like Klemun’s, recognize that there is room for decentralizing the systems through which we assess SDG achievement by presenting an alternative to global snapshots of “synergies and trade-offs.”^35^ The Agenda calls for the implementation of the SDGs at the subnational and local levels, yet does not develop or recommend specific instruments to facilitate the process. Frameworks like Klemun’s show that it is possible to make SDG-compliant and quantitatively robust choices at a granular level of decision-making as well. Conversely, these instruments can also be used to filter out ineffective technologies or interventions. It is essential for the UN Agenda 2030 to not remain impartial to the development of such quantitative tools but rather incorporate them in its structure and encourage their refinement.

3. Greater emphasis on de-risking sustainability investments

When faced with the choice of supporting sustainable innovations, marketability and cost have been of exclusive importance to policymakers, often to the detriment of other dimensions embodied in the Agenda. While the cost of PV systems has fallen considerably since 2012, race and income disparities in their diffusion remain stark^39^ and indicative that the climate action story has only penetrated select communities, falling short of climate justice aims. Income disparities are notable between nations as well, with clusters of countries specializing in particular SDGs, while simultaneously creating “orphaned” SDGs that lag behind.^40^

We propose a mission-oriented approach for de-risking sustainability investments by governments and international bodies in which pecuniary motivations play a secondary role.^41^ This mission-oriented approach underlies the success of Germany’s feed-in-tariff, which transformed its market into the world’s largest for solar PVs. The UK’s contract-for-difference auctions that have unlocked renewable energy investments throughout the country follow the less successful Renewables Obligations (RO) regime, which increased competition between generators but also heightened investors’ risk,^42^ offering a cautionary tale against a pure market approach in the early diffusion stages.

As scholars have pointed out, the role of markets in the subtle transition period to net zero is limited given the fundamental mismatch between sectors whose emissions are most valuable to society and those whose emissions are most costly to remove,^43^ strengthening the argument for putting social and environmental needs first during this time. A mission-driven approach to climate financing can ensure that communities are neither relegated to the margins of adaptation and mitigation efforts nor have their interests overridden by donors’ priorities.^44^ Empirical studies have also shown that justice-centered and decentralized approaches to early-stage sustainable solutions encourage the development of local businesses that further facilitate the diffusion of innovative technologies in underrepresented communities.^39^

4. Implementing decentralized solutions by rewarding social innovation

A successful decentralized approach to the SDGs hinges on recognizing and rewarding social innovation. However, such recognition has consistently lagged behind that of scientific breakthroughs. There exist both long-established and new prizes (e.g., the Breakthrough Prize) to reward technological innovation and basic science progress in the natural sciences. Similarly, national-level policies have been formulated to aid the diffusion of sustainable technology, from rooftop PVs to electric vehicles and to assist their scholarly research. The same cannot be said for social initiatives.

Closing these sustainability gaps requires recognizing and rewarding individual and community efforts that extend sustainability initiatives beyond a limited set of countries and communities. Research output on the SDGs abounds in examinations of member countries of the Organisation for Economic Co-operation and Development (OECD) while less attention has been devoted to sustainability efforts in other regions.^45^ Context-specific solutions can be encouraged by establishing prizes and fellowships that recognize the transformational potential of social progress. Institutional recognition of promising initiatives would facilitate the exchange of expertise for designing and spreading tailored solutions to previously intractable problems, such as water-saving irrigation methods in drought-prone contexts^46^ or mass-manufacturable medical devices^47^ that can be easily deployed in industrializing nations. This is all the more reason for encouraging decentralized interventions that do not have to depend on convoluted top-down decision-making but rather can rely on successful localized trials, be implemented promptly, and benefit from local acceptance and know-how.

Strong incentives and rewarding mechanisms for innovative social interventions can benefit the SDGs, but such support remains only half the story. On the implementation side, responsive institutions need to deploy rigorous means of identifying and scaling promising interventions and also develop capacities to anticipate their amplifying positive impact as well as the obstacles that they could pose for the goals. There is no shortage in the literature of failed solution attempts that were flimsy on the social intervention side.^48^^,^^49^ They often include shortcuts in implementing top-down programs rather than taking the more tiresome path of identifying locally successful initiatives and supporting their scale-up. These examples support the thesis that rewarding social innovation and integrating its best examples in institutional contexts not only advances overlooked social SDGs but also is an excellent medium to spur progress across all the goals.

## Conclusion

**Figure 2:**
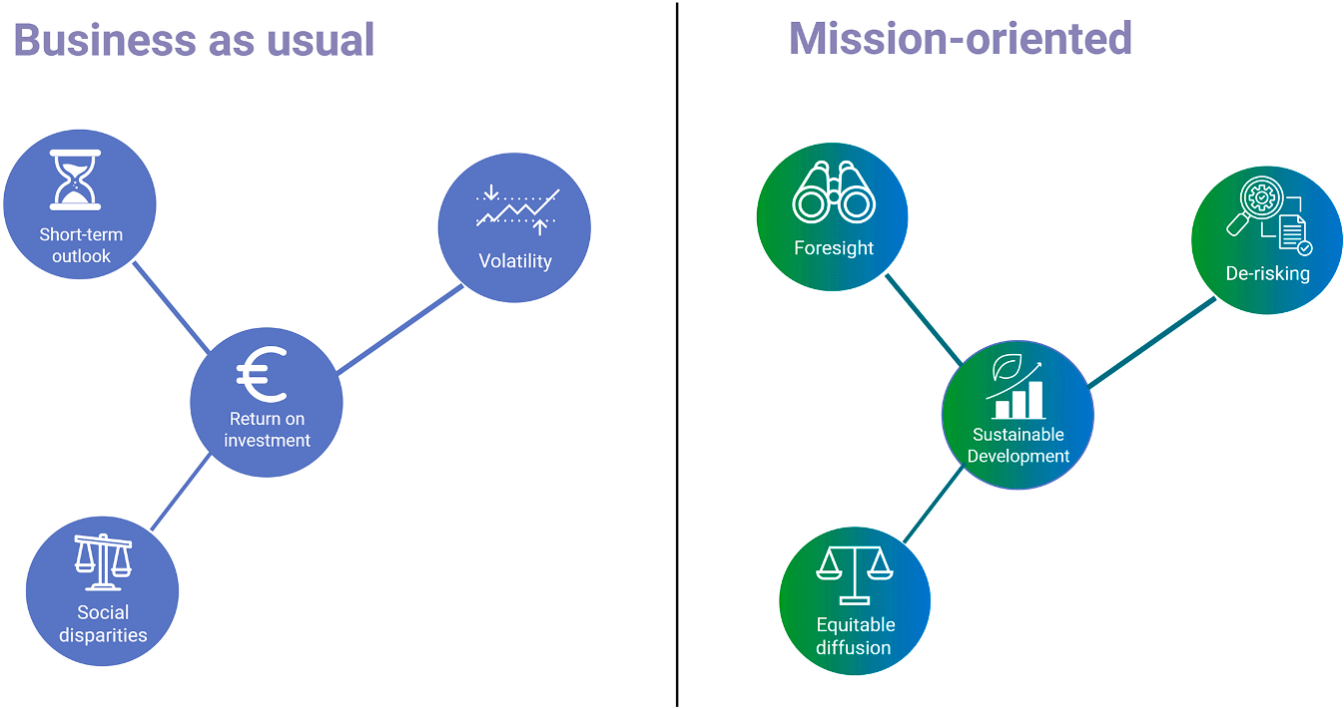
Areas of focus under the traditional and proposed approaches to climate solutions

With the UN Agenda’s 2030 deadline looming and dramatic anti-sustainability stances in some major economies, the shortfalls in the fulfillment of the SDGs call for a rethinking of the UN Agenda’s purpose and structure (Figure 2). We see the upcoming COP 2026 and the attention it garners as an opportunity to examine the SDGs and the overarching framework of the UN Agenda. Given the global-scale disruptions of recent years in the form of a pandemic, trade shifts, and fast technology developments, it is essential for the UN Agenda to become a “living document” by including clauses that require its periodic review. Keeping up with the times also demands the development of rigorous quantitative methods and a common language to refer to SDG interlinkages, a domain whose study remains inconclusive and, as a result, hinders the sharing of best practices in advancing the goals. The need is dire for best practices to spread widely across communities, especially during the current mid-transition period where sustainable systems are not firmly established yet the disruption arising from their adoption is visible. For this reason, we advocate that the Agenda meet the moment and recognize the secondary importance of financial factors in helping to launch innovations that ensure progress in the societal, health, justice, and environmental realms. Driven by this mission, promising net-zero innovations should be de-risked and societally relevant interventions should be encouraged.

Given the stalling progress of countries toward end targets, it is high time for the Agenda to cease relying solely on the goodwill of signatories and also introduce enforceable progress measures. Our analysis asserts that a reason for the limited advancement of SDG targets is the little attention devoted to decentralization in the Agenda, with states expected to play the main role in launching and steering goals. As recent examples show, top-down governmental measures are not guaranteed to work, and there is much potential in the institutional uptake of successful decentralized approaches in all areas of sustainability. This perspective outlines a suite of linked recommendations in these three areas to ensure that more SDGs are on track to meet their targets while also laying the groundwork for a post-2030 Agenda that is firmly grounded on bridging the gap between aspirations and implementation.

## Acknowledgments

This work was partly funded by the German Federal Ministry of Research, Technology and Space (BMFTR) under grants 03SF0626C (WASCAL IMP-EGH) and 03ZU2115KA (HyInnoSys 2). The authors would like to thank the German Committee Future Earth (DKN) for hosting the Sustainability Science Summit in Berlin in February 2025. A session jointly organized by the authors of this manuscript at this event served as the starting point for the research efforts that led to this manuscript.

## Author contributions

All authors jointly developed the research concept and contributed to the conceptualization of the first draft. R.S. wrote the initial draft of the article and rewrote portions of the article after the review rounds. D.M.K. reviewed and edited the initial draft. M.K. reviewed and edited the initial draft and reviewed and rewrote portions of the article after the journal revision rounds. S.V. organized and hosted the Berlin session, reviewed and edited the initial draft, and reviewed and edited the final draft.

## Declaration of interests

The authors declare no competing interests.

## Declaration of generative AI and AI-assisted technologies in the writing process

No AI software was used.
